# Fabrication and Characterization of Thermal-Responsive Biomimetic Small-Scale Shape Memory Wood Composites with High Tensile Strength, High Anisotropy

**DOI:** 10.3390/polym11111892

**Published:** 2019-11-15

**Authors:** Luhao Wang, Bin Luo, Danni Wu, Yi Liu, Li Li, Hongguang Liu

**Affiliations:** MOE Key Laboratory of Wooden Material Science and Application, Material Science and Technology College, Beijing Forestry University, Beijing 100083, China; 13269302606@163.com (L.W.); 15393366580@163.com (D.W.); liuyichina@bjfu.edu.cn (Y.L.); lili_email@263.net (L.L.); liuhg_liuhg@126.com (H.L.)

**Keywords:** shape memory wood, thermal-response, biomimetic materials, interface bonding

## Abstract

Intelligent responsive materials have become one of the most exciting fields in the research of new materials in the past few decades due to their practical and potential applications in aerospace, biomedicine, textile, electronics, and other relative fields. Here, a novel thermal-responsive biomimetic shape memory wood composite is fabricated utilizing polycaprolactone-based (PCL) shape-memory polymer to modify treated-wood. The shape memory wood inherits visual characteristics and the unique three-dimension structure of natural wood that endows the shape memory wood (SMW) with outstanding tensile strength (10.68 MPa) at room temperature. In terms of shape memory performance, the shape recovery ratio is affected by multiple factors including environment temperature, first figuration angle, cycle times, and shows different variation tendency, respectively. Compared with shape recovery ratio, the shape fixity ratio (96%) is relatively high and stable. This study supplies more possibilities for the functional applications of wood, such as biomimetic architecture, self-healing wood veneering, and intelligent furniture.

## 1. Introduction

Wood is a natural renewable material that has been widely used in all works of life due to the excellent mechanical properties and friendly environment characteristics. Compared with plastics, steel, cement, and other structural material, wood has unique mesoporous and hierarchical structure stemming in its biological origin, which makes it inherit wood material characteristics such as high ratio of strength to weight, certain viscoelasticity, and anisotropy. The mesoporous structures that consist of pores in hardwood, lumina varying in diameter from 10 to 80 μm, pits, and gum canal or resin canal, which together form the interpenetrating cannular networks and offers the penetrability, accessibility, and reactivity of wood materials [1,2,3]. In the past few decades, bio-inspired intelligent responsive materials have become one of the most exciting fields in the research of new materials. Advanced stimuli-responsive wood materials have been made by taking advantages of wood, such as reversible pH-responsive material [4,5], wood fiber gas sensor [6], light-responsive wood functional material [7,8,9], thermal-responsive wood-based hydrogel, and color-change material [10,11].

Shape-memory polymers (SMP), one type of smart materials, can deform from the temporary shape to the permanent shape under external stimuli (mainly including heat [12,13], light [14], electric [15], magnetic fields [16], water/solvent [17]), where performance had been programmed before triggering [18,19], and that it has common advantages of polymer: High elastic deformation, low cost, low density, potential biocompatibility, and biodegradability. Indeed, SMP has been widely used in aerospace [20], textile [21], biomedicine [22], kinetic buildings, and other related fields [23]. In order to improving SMP’s physical and chemical properties, one of methods is by introducing filler into polymer matrix, such as wood flour and cellulose whisker [24,25], which all have destroyed the original biological structure and morphology of natural wood. This work doesn’t aim to improve the performance of shape memory polymer, but focus on functionalized modification and biomimetic research of natural wood.

During the last decade, self-shaping wood was fabricated by bilayer cross-laminated structure design, which can change the curvature of large-scale mass timber on the meter scale by changing wood moisture content (WMC) [26,27,28]. This self-shaping occurs as a result of the different expansion ratios of wood in transverse and parallel wood grain directions, when changing WMC by either drying or wetting, and that it’s shaping process is continuous without fixed threshold value, therefore it has no shape memory ability. Compared with self-shaping wood, this research is capable of self-shaping on the centimeter scale, and memory the temporary shape until reaching stimuli-condition.

Due to the mesoporous structure deriving from biological growth, wood can be used as a carrier to make wood composite material, such as thermal-responsive wood hydrogel and wood sponge [29], which all preserve intact multi-scale porous structure and visual characteristics. More importantly, intact mesoporous structure makes wood have a higher ratio of strength-to-weight, and strong tensile strength parallel to grain of wood, which endows wooden-composites with outstanding mechanical properties. For instance, wood hydrogel composite that was synthesized through polyacrylamide (PAM) hydrogel not only exhibited characters of common hydrogels, but also displayed a high-tensile strength of 30 MPa on longitudinal direction (500 times stronger than unmodified PAM hydrogel) [30]. These studies offer a promising laboratory method to combine wood with SMP. The aim of this study is to fabricate shape memory wood (SMW) composite, where the original channels in wood align perpendicularly to the wood lengthwise direction. We investigated the process-mechanism-properties of SMW, and found that SMP was well injected into the mesoporous structure in wood, and formed a cross-linking system. The resulting SMW composite maintain the micro-structures and visible characteristics of wood, and that the mechanical tensile strength is significantly higher than of the original wood. For the first time, we tentatively fabricated and verified the shape memory wood composite without systematic study on the effect of weight concentration and cross-linking density of SMP on the properties of SMW, which will be systematically researched in the follow-up work.

## 2. Materials and Methods

### 2.1. Materials and Chemicals

The oligo(ε-caprolactone) diols (o-PCL) with number average molecular weight (*Mn*) of 2000 was purchased from Hubei Chushengwei Chemical Co., Ltd. Glycidyl POSS (Merchandise sign Hybrid Plastics EP0409, hereafter referred to epoxy-POSS, Changsha, China) was viscous liquid at room temperature, and supplied by Hybrid Plastics Inc. The balsa wood (*Ochroma lagopus*, Heze, China) was obtained from Cao County Tongtong Wood Industry Co., Ltd. Sodium hydroxide (AR, Aladdin, Shanghai, China), sodium sulfite (AR, Aladdin, Shanghai, China), tetrahydrofuran (THF, >99.0%, Macklin, Beijing, China), ethanol (AR, Macklin, Beijing, China), anhydrous magnesium perchlorate (Mg(ClO_4_)_2_, AR, Macklin, Beijing, China) and deionized (DI) water were used as received.

### 2.2. The Process of Delignification

The balsa wood block was incised to several thin slices with a dimension of 80 mm(length) × 10 mm(width) × 2 mm(thickness), where wood channels are perpendicular to the thickness plane direction. Above test specimens were immersed in delignification solution that consisted of NaOH (2.5 mol/L), Na_2_SO_3_ (0.4 mol/L) in DI water, and the solution was heated to boiling and stirred continuously for 24 h. These test specimens were then immersed in hot DI water and carefully rinsed for several times to remove residual chemicals until the hot DI water was neutral. Finally, these lignin-removed wood (LRW) samples were put in vacuum freeze-dryer at −45 °C for 48 h after frozen at −20 °C for 12 h to remove residual DI water and obtain good wood mesoporous structure.

### 2.3. Preparation of Impregnation Polymer

Star-epoxy-POSS with eight same arms; every arm has an epoxy group in a terminal, which all have the potential to react with hydroxy of o-PCL by ring-opening reaction. Dissolving o-PCL, epoxy-POSS, Mg(ClO_4_)_2_ in THF solvent according to molar ratio of o-PCL/epoxy-POSS 4:1 and weight ratio of Mg(ClO_4_)_2_ 0.2% of total reactant, the mixture solution was then vigorously stirred by a magnetic stirring apparatus for 12 h at room temperature until obtaining homogeneous solution. Finally, the homogeneous solution was put into vacuum spin evaporator and degassed under −0.09 Mpa to evaporate THF solvent bathing in 60 °C hot water until no bubbles could be observed.

### 2.4. Preparation of the Shape Memory Wood

Placing the LRW samples in a Teflon dish, the solvent-removed homogeneous solution was poured into the dish and completely covered the surface of these samples, and after that, the Teflon dish was placed in the vacuum drying oven and kept at constant temperature (80 °C) to maintain the solution in a viscous state. The vacuum drying oven was degassed to below −0.098 MPa and kept that vacuum degree for 10 min. Then, releasing the vacuum valve, the homogeneous solution was forced to impregnate into the wood porous structure (mainly including the lumen, cell wall, and other intercellular space) by atmosphere pressure, keeping that state for 20 min. All the above steps were repeated several times (4–6) until no obvious bubbles could be observed on the surface of wood samples. When finishing the above impregnation process, residual viscous solution sticking on the surface of wood samples was cleaned. Finally, the wood samples were heated to 135 °C in the vacuum drying oven for 4 h. After o-PCl and epoxy-POSS were completely cured, the samples were taken out of the vacuum drying oven, stored in the desiccator, and named as shape memory wood (SMW).

### 2.5. Measurements and Characterization

The wood morphological structure of natural wood, LRW and SMW were observed using the scanning electron microscope (SEM, JEOL JSM-6700F, Tokyo, Japan). Grinding LRW, SMW, and crosslinking-PCL (c-PCL) into small particles and sealing in a metal dish, after that using differential scanning calorimeter (TA Instruments, TA DSC Q2000, New Castle, DE, USA) analyzed thermal properties of these samples. The temperature was from −30 °C to 130 °C, with a rate of 10 °C min^−1^, and included two heating stages and one cooling stage, in which the first heating stage would eliminate heat history of samples. Attenuated total reflectance–Fourier transform infrared spectroscopy (Thermo Fisher, ATR-FTIR Nicolet 6700, Waltham, MA, USA) supplied the FTIR information and analysis. The dynamic thermomechanical properties of c-PCL and SMWs were tested by DMA (TA Instruments, TA Q800, New Castle, DE, USA) with a frequency of 1 Hz, and the temperature was from −30 °C to 130 °C with a heating rate of 5 °C min^−1^. The XPS apparatus (Thermo Fischer, ESCALAB 250Xi, Waltham, MA, USA) with Al Kα ray (λ = 1486.6 eV) was used to investigate the elements changes of LRW and SMW before and after impregnation, which performed with a 40 eV passing-energy and used the C 1s = 284.60 eV as energy standard for charge correction.

The weight concentration of SMP (*W_cSMP_*) in SMW was calculated as $W_{rSMP}=\left[\frac{m-m_{0}}{m_{0}}\right]\times 100\%$, by choosing 5 samples from the same batch to weigh the initial weight m_0_ and the weight after impregnation m, respectively. The gel content was measured by Soxhlet extraction to investigate the crosslink density of networks, and defined as Gel (%) as follows. The SMW, c-PCL, LRW were extracted for 48 h in hot THF (~66 °C) after weighing the initial weight, then, they were dried to constant weight. The residual weight of SMW includes the mass loss of LRW.

$$\mathrm{Gel}\;\left(\%\right)=\frac{weight\;of\;residue\;\left(g\right)}{initial\;weight\;\left(g\right)}\times 100\%$$

Randomly choosing three LRW samples, to reckon the average porosity of LRW based on Archimedes drainage method principle, the formula $P_{LRW}=\frac{m_{2}-m_{0}}{m_{2}-m_{1}}\times 100\%$ was used, in which m_0_ is the oven-dried weight of LRW, and m_2_ is the weight of LRW that were immersed in DI water for 144 h until reaching maximum moisture content of LRW, and m_1_ is the suspending weight of LRW totally immersed in DI water at room temperature. The density of PCL&POSS mixture solution (PP) were determined using mass–volume method, keeping molten state at 80 °C. The volume filling ratio of SMP (*V_rSMP_*) in SMW was defined as *V_rSMP_* (%), as follows:

$$V_{rSMP}\;\left(\%\right)=\frac{m_{pp}}{V_{SMW}\times {\;\bar{P}}_{LRW}\times \rho_{pp}}\times 100\%$$

**Figure 1:**
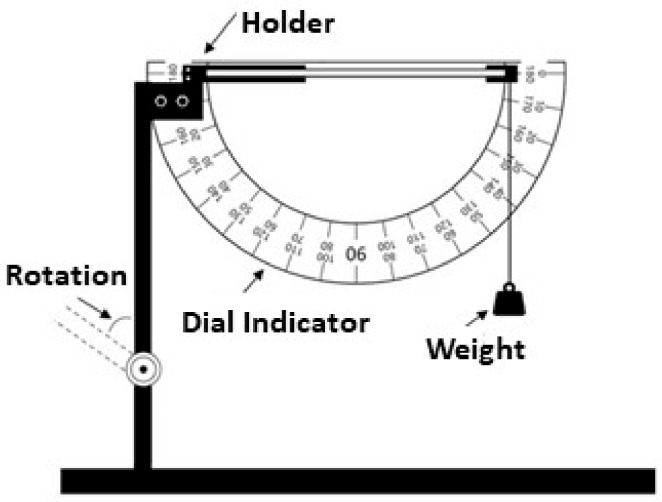
Angle measurement device for testing the shape memory properties of materials.

The shape memory properties of SMWs were investigated along cross-section direction using a goniometer fixed on a holder with a specific gap that was used to clamp the samples, as shown in Figure 1. This device was put in a heating box with a temperature heating and control system, and the deformation process was recorded by a high definition camera. Shape recovery ratio (*D_r_*) and shape fixity ratio (*D_f_*) in different heat condition were calculated by the below formula:

$$D_{r}\;\left(\%\right)=\frac{\beta_{d}-\beta_{r}}{\beta_{d}-\beta_{i}}\times 100\%$$

$$D_{f}\;\left(\%\right)=\frac{\beta_{f}-\beta_{i}}{\beta_{d}-\beta_{i}}\times 100\%$$

*β_i_*: The initial bending angle degree of SMWs.
*β_d_*: The deformation bending angle degree of SMWs in external force.
*β_f_*: The bending angle degree without external force at room temperature.
*β_r_*: The residual bending angle degree in recovery process at different experiment condition.

## 3. Results and Discussion

**Figure 2:**
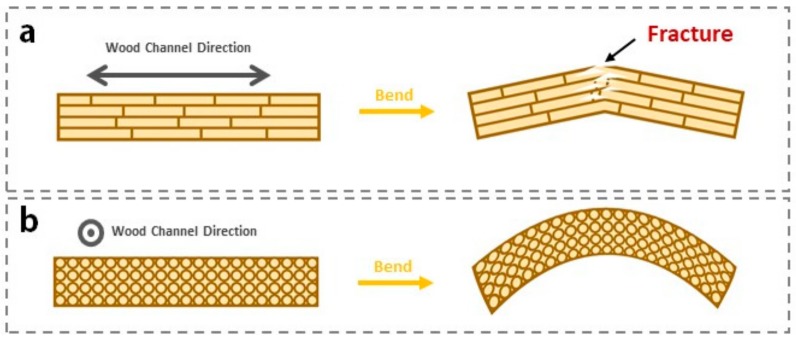
Graphical illustration showing two different direction of wood. (**a**) Bending deformation along wood channels direction. (**b**) Bending deformation along the direction perpendicular to the wood channels.

Natural wood is kind of heterogeneous, anisotropic, and macromolecule material whose mechanical properties are different from other materials, showing up viscoelasticity and creep characteristics to some extent. Depending on the glassy transition of lignin, natural wood also has a certain shape memory effect, where temperature control and moisture content are essential for fixing natural wood deformation. For another, the compression of cell lumen and the slippage of cellulose fibers immensely weaken the shape memory properties of natural wood [31]. In this study, the anisotropic of wood mainly reflects in the distinction of shape memory performance along different bending direction. In Figure 2a, wood channels aligned along cross-section direction will cause stress concentration on the bending place during deformation stage, and then stress fractures the cell wall structure of wood, resulting in the disappearance of shape memory effect in SMW. This is the reason we choose cross-section direction as bending direction to survey shape memory properties.

### 3.1. Morphology of the Shape Memory Wood

**Figure 3:**
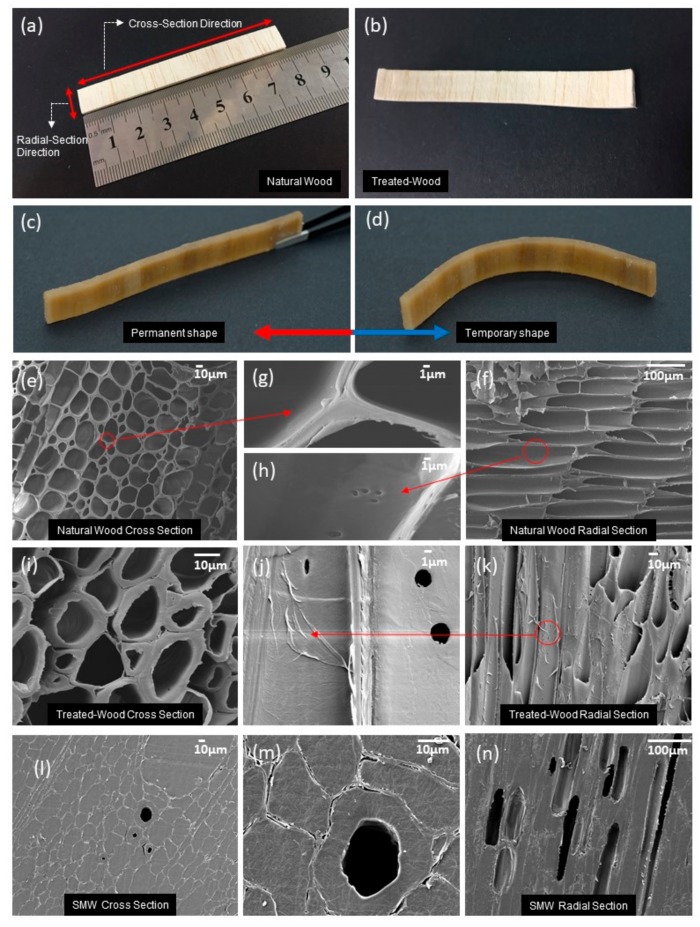
Optical and SEM image of natural wood, treated-wood, and shape memory wood (SMW): (**a**) Natural wood. The balsa wood veneer was cut along cross-section direction that is perpendicular to the length of wood fiber. (**b**) Treated-wood. (**c**) The permanent shape of SMW. (**d**) The temporary shape of SMW after figuration. (**e**) SEM image of natural wood along cross-section direction. (**f**) SEM image of natural wood along radial section direction. (**g**) True middle lamella SEM image of natural wood. (**h**) Cell wall SEM image of natural wood. (**i**) SEM image of treated-wood along cross-section direction. (**j**) Cell wall SEM image of treated-wood. (**k**) SEM image of treated-wood along radial section direction. (**l**) SEM image of shape memory wood along cross-section direction. (**m**) Detail lumen SEM image of shape memory wood. (**n**) SEM image of shape memory wood along radial section direction.

Figure 3 shows the internal micro-sized structure of natural wood, LRW and SMW. We found that the three-dimension mesoporous network structure had been preserved after delignification treatment, and some obvious changes had arisen in the cell wall and true middle lamella. The secondary cell wall of natural balsa wood is smooth in cross-section direction, and no peeling or delamination can be observed in radial section direction in Figure 3e–h. However, after delignification process, the secondary cell wall had already occurred distinct peeling phenomenon in the inner layer of cell wall, and true middle lamella was delaminated as observed in Figure 3i,j. All these changes are prerequisites to achieve shape memory performance.

According to the results of *W_cSMP_* and *V_rSMP_* in Table 1, it was seen that the average *W_cSMP_* and *V_rSMP_* in SMW can reach to 555% and 86.54%, respectively. In Table 2, the gel content of SMP in SMW is about 56.83%, which was obviously lower than 61.53% of pure c-PCL. This indicates that LRW scaffold has a certain negative effect on the cross-linking reaction of PP. In terms of dimensional changes, compared with LRW, the shape volume of SMW expanded by about 5.44%, especially in the thickness direction, as shown in Table 1, which can prove that PCL molecular chain diffuses into the cell wall of LRW scaffold and results in its swelling.

### 3.2. The Combination Mechanism and Crosslinking

**Figure 4:**
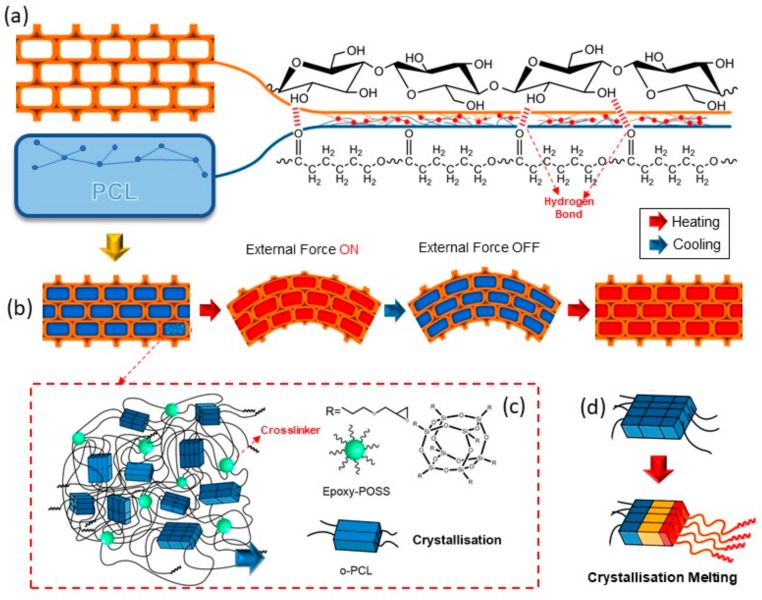
Schematic illustration showing the combination mechanism of shape memory wood. (**a**) Schematic to illustrate the combination between wood and PCL, which depicts the generation of hydrogen bonding between -OH and C=O. (**b**) Graphical depiction of SMW deformation process under external force at heating and cooling condition. (**c**) Graphical illustration of covalent cross-linking structure between o-PCL chains and depiction of crystal distribution that endows SMW transition temperature and rigid fixation. (**d**) Graphical depiction of crystallization melting process.

**Figure 5:**
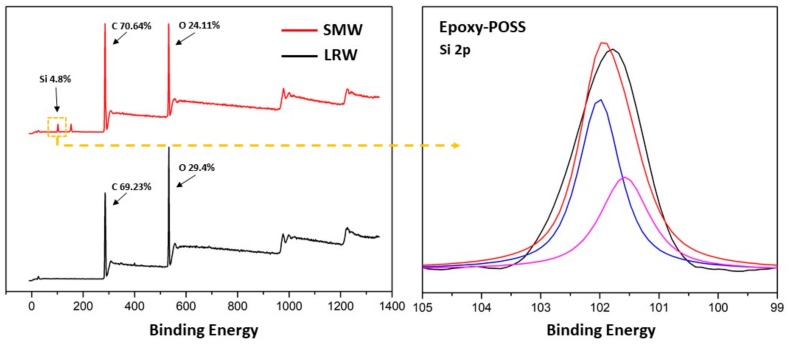
X-ray photoelectron spectroscopy scans of LRW and SMW.

**Figure 6:**
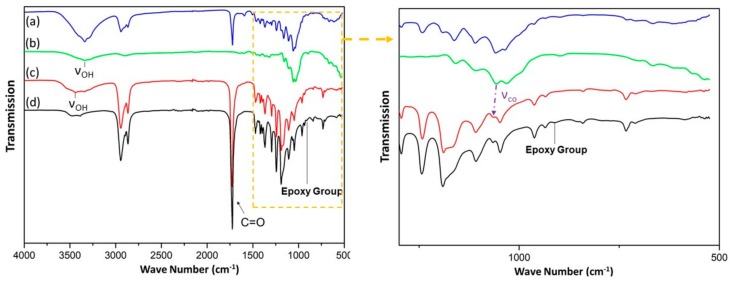
FTIR Spectra: (a) Natural wood; (b) lignin-removed wood(LRW); (c) shape memory wood(SMW); (d) PCL & epoxy-POSS mixture solution(PP).

In Figure 4, the proposed graphical illustration explains the combination mechanism between PCL and wood, and graphically depicts the deformation process of SMW. As is known to all, natural wood has throughout three-dimension mesoporous network structure, which makes PP easy to penetrate the voids of wood (lumina, pits, intercellular space, and so on) under atmospheric pressure. In addition, lignin-removed process softened wood cell wall structure and expanded the inner space of wood, and make cellulose fibers have more opportunities to interact with PCL molecular chain. Figure 4a illustrates the mutual effect mechanism between the wood and PCL molecular chain, where graphically indicate the generation of hydrogen bonds between O–H and C=O, and which endows shape memory wood outstanding interface bonding strength and excellent stability. In Figure 5, the Si 2p peak around 102 eV in the spectrum of SMW demonstrated epoxy-POSS had been introduced into wood [32]. Then, the oligo(ε-caprolactone) diols can crosslink with the mutual functional group by the reaction between hydroxyl group (o-PCL) and epoxy group (crosslinker, epoxy-POSS) in proper reaction conditions, and the reaction product named as c-PCL (Figure 4c) [33]. We performed Fourier Transform Infrared Spectroscopy (FTIR) to investigate the crosslinking reaction between o-PCL and epoxy-POSS, and the generation of hydrogen bonds between wood and PCL (Figure 6). On the FTIR spectra of Figure 6d, the peak at 911 cm^−1^ indicates the absorption of the epoxy group. After high-temperature curing process, this peak disappeared on the FTIR spectra of “SMW” in Figure 6c, which represents the polymerization reaction between o-PCL and epoxy-POSS. Additionally, on the FTIR spectrum of LRW (Figure 6b), the peaks at 3336 cm^−1^ and 1056 cm^−1^ correspond to the O–H stretching and C–OH stretching, respectively [30,34], and on the FTIR spectrum of PP (Figure 6d), the peak at 1724 cm^−1^ corresponds to the C=O stretching. After impregnation and curing process, the peak position of O–H and C–OH correspondingly shifted from 3336 cm^−1^(LRW) to 3437 cm^−1^ (SMW) and from 1056 cm^−1^ (LRW) to 1109 cm^−1^ (SMW), and the peak at 1724 cm^−1^(PP) increased to 1726 cm^−1^ (SMW). The blue shift of above functional groups in FTIR spectrum demonstrated the formation of hydrogen bonds between O–H and C=O [35].

### 3.3. Thermal and Mechanical Properties

**Figure 7:**
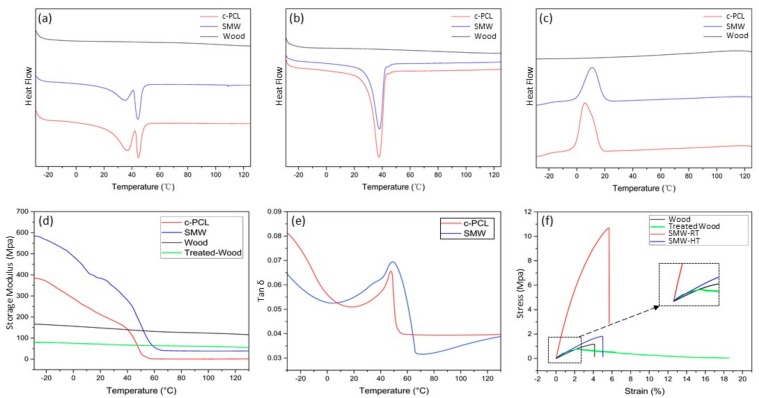
DSC curves of c-PCL, SMW, and wood: (**a**) First heating stage; (**b**) second heating stage; (**c**) cooling stage; DMA curves of c-PCL, SMW, wood, and treated-wood; (**d**) storage modulus; (**e**) tanδ curves of c-PCL and SMW; (**f**) tensile stress-strain curves of wood, treated-wood, SMW-RT (room temperature), and SMW-HT (temperature 65 °C).

Thermal properties of natural wood, treated-wood, c-PCL, SMW were investigated by DSC, DMA. Using differential scanning calorimeter (DSC), we investigated the transition temperature and enthalpy change of natural balsa wood, SMW, c-PCL, respectively. The sharp melting transition and the crystalline transition were observed in Figure 7, which all act as a switch to control the shape memory behavior and endow wood with shape memory ability in thermodynamics. Interestingly, in the first heating stage of DSC test, the curves of c-PCL and SMW display distinct double melting peaks in Figure 7a, which differ from the second heating stage in Figure 7b that just display one melting peak. This characteristic could be ascribed to the rearrangement of the crystal layer that originally neatly arranged in c-PCL phase. After the preparation process of DSC samples, these crystal layers were destroyed, and double melting peaks appeared, in which lower melting peak represents the initial liquation and the higher melting peak represent the second liquation [36,37]. The second heating curves were shown in Figure 7b, where c-PCL and SMW have almost the same melting temperature (*T_m_*), about 37.8 °C, but the melting enthalpy of SMW (*ΔH_m_* 35.06 J/g) is significantly lower than c-PCL (*ΔH_m_* 41.31 J/g). We consider the reduction of melting enthalpy is mainly because of the effect of wooden components in SMW, which not only reduce the relative content of c-PCL, but also do not have obvious melting enthalpy. Additionally, as shown in the cooling stage curves (Figure 7c), the crystalline temperature (*T_c_*) of SMW (about 11.14 °C) is distinctly higher than c-PCL, whose *T_c_* is 5.61 °C, but crystalline enthalpy of SMW (*ΔH_c_* 37.26 J g^−1^) is obviously lower than c-PCL (*ΔH_c_* 42.76 J g^−1^) due to the same reason as above. Furthermore, we noticed a slight increase of SMW’s *T_c_* in Figure 7b. This may be a result of the existence of tiny wood particles that were produced in the preparation process of test sample and could act as a nucleating agent to promote the crystallization of PCL.

Shape memory wood sample also possesses certain viscoelastic properties as c-PCL, which can be proved by tan δ data using dynamic thermomechanical analysis (DMA). In Figure 7e, we can find an obvious tan δ peak of SMW ranges from 40 °C to 55 °C, which is in accordance with the temperature interval of storage modulus sharp drop that is through the temperature region from 35°C to 55 °C in Figure 7d. The sharp drop of storage modulus also identifies T_m_ of SMW and c-PCL, which is in good agreement with DSC. On the other hand, although SMW and c-PCL had shown similar linear trends, the storage modulus of SMW is significantly higher than that of c-PCL. There are two main reasons for the increased storage modulus, one is the increase of crystallinity as discussed above, and the other is the influence of wood porous structure that improves the overall strength of SWM.

Mechanical strength is a significant factor for shape memory wood composite to be used in practical applications. As we all know, the included angle between the tensile direction and the wood texture direction is one of the most significant factors affecting the strength of wooden material [38]. The tensile strength perpendicular to the grain of the wood is usually only 1/65–1/10 of the tensile strength parallel to the grain of the wood. Therefore, in this study, we investigated the tensile strength of SMW, treated-wood, and natural wood along the cross-section direction at room temperature. In order to observe the change of tensile strength of SMW before and after melting temperature, a heating cabinet was used to heat the shape memory wood sample to 65 °C.

In Figure 7f, we unexpectedly find that the tensile strength of treated-wood is higher than natural wood in the initial stretching stage (strain 0%–2%). One possible explanation is the delignification process reduced the interval between microfibril by removing lignin and hemicellulose, and which may increase cell wall density and crystallinity of the cell wall. These changes consequently enhanced the tensile strength of treated-wood. Attractively, as can be seen from Figure 7f, the tensile strength of SMW-RT (10.68 MPa) is 805% higher than natural wood, which reflects the strong interface bonding between wood and c-PCL. Furthermore, when the temperature was above *T_m_*, the tensile strength of SMW-HT sharply declined, but it’s still higher than that of natural wood and close to the tensile strength of treated-wood. The excellent tensile strength is beneficial to exaggerate the application range of SMW, such as intelligent furniture, wooden self-actuating device.

### 3.4. Shape Memory Properties of Shape Memory Wood

**Figure 8:**
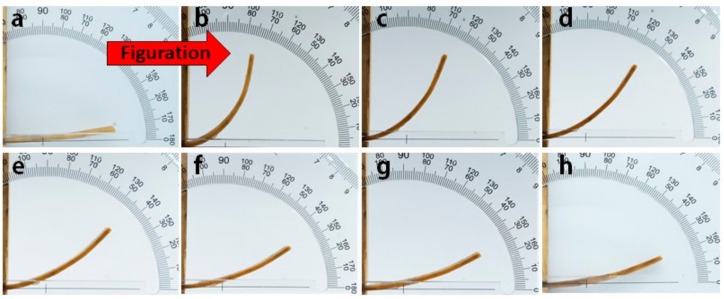
Optical images of SMW deformation process: (**a**) Initial shape of SMW; (**b**) temporary shape of SMW; (**c–h**) the deformation process of SMW.

**Figure 9:**
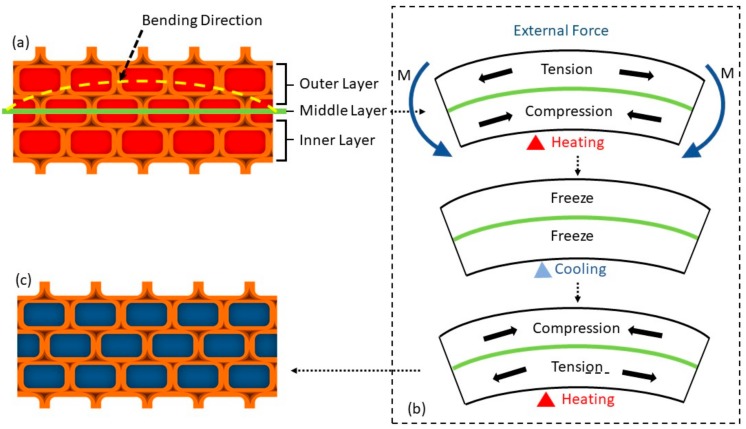
Schematic shape memory mechanism of SMW during deformation under external condition: (**a**,**c**) Graphical illustration of SMW’s structure on cell level; (**b**) graphical depiction of inner stress of SMW on deformation process.

Shape memory properties including shape fixity ratio, shape recovery ratio, cycle shape memory ability, and other shape memory properties were investigated by an angle measurement method. In order to fundamentally understand the shape memory process of SWM as shown in Figure 8, we proposed the shape memory mechanism of SMW according to the synthetically analysis of SMW thermodynamic behavior in Figure 9. Natural balsa wood has a well-developed, three-dimension mesoporous structure that is mainly composed of the relatively independent lumen, vessel, fibrous tracheid, axial parenchyma, and xylem ray. After the wood was impregnated, these lumina were consequently filled by c-PCL, as shown in Figure 9a,c. When the treated-wood is bent, the outer layer lumina will bear tension stress, and the inner layer will sustain compression stress, both stress directions are parallel to the longitudinal direction of SMW. As a result, the outer layer lumina will be stretch, meanwhile, the inner layer will be compressed. These changes in wood structure will compel intracellular c-PCL to generate morphologic change when the temperature is above *T_m_*, and the shape of SMW will then be fixated to temporary shape when lowering the temperature to *T_c_*. On the contrary, when the temperature rises, the SMW will recover to its permanent shape, driven by entropic elasticity of c-PCL.

**Figure 10:**
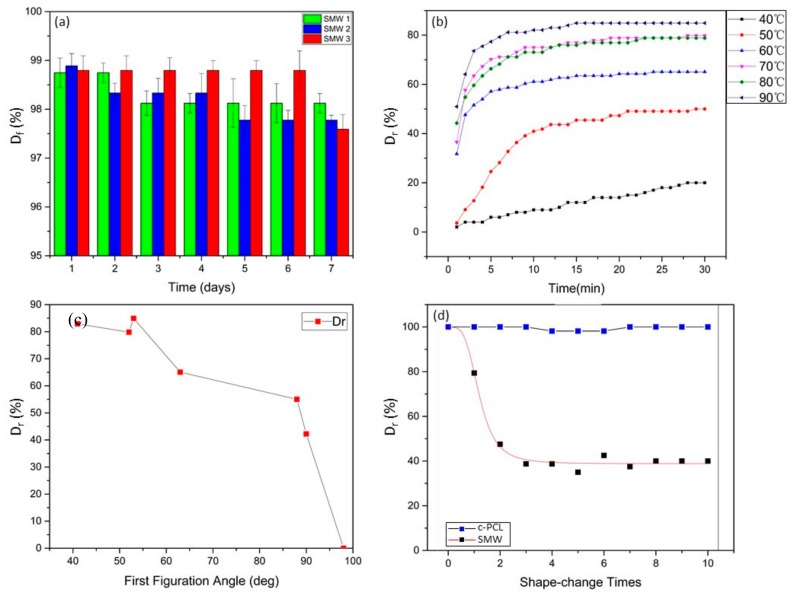
Shape memory properties of shape memory wood: (**a**) Relationship between shape fixity ratio (*D_f_*) and time of SMW; (**b**) relationship between shape recover ratio (*D_r_*) and time of SMW in different heating temperature; (**c**) relationship between shape recover ration and first figuration angle degree of SMW; (**d**) relationship between shape recover ration and shape-change times of SMW, c-PCL.

**Figure 11:**
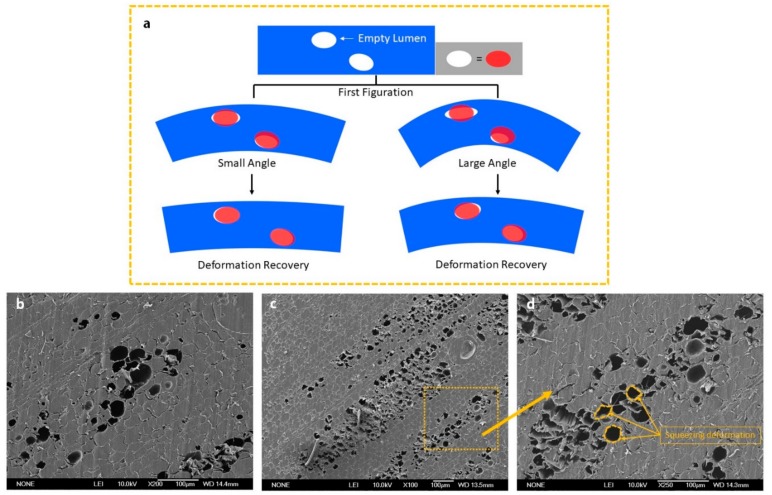
(**a**)Graphical illustration of the unrecoverable residual deformation in SMW, where large bending angle results in an increase of residual deformation; (**b**) the SEM image of empty lumina before bending deformation; (**c**,**d**) the SEM images of empty lumina after bending deformation.

The shape fixity ratios were tested within one week at room temperature, using three different SWM samples that had same dimensions, and were fabricated under same processing conditions, and the results are shown in Figure 10a. Compared with the shape recovery ratio in Figure 10b, the shape fixity ratio is relatively stable, which all three groups of SMWs were above 96% during the test period. In order to obtain insight into the decrease of the shape recovery ratio, we implemented SEM measurements to research the inside variation of SMW after several deformation cycles. Through the analysis of SEM image in Figure 3l–m and Figure 11, we thought the existence of empty lumina scattered inside SMW may decrease the shape recovery ratio. Because wood is a kind of plastic material, which will preserve a currently unrecoverable deformation when the stress exceeds the elastic limit of the wood. The residual deformation of wood will form a reactive force to resist entropic elasticity force produced by PCL molecular chain, and the shape recovery ratio of SMW would decrease at the same time. In addition, these empty lumina could lead to the gradually extending separation of the cell walls in the vicinity, as shown in Figure 3m, which further decrease the shape recovery ratio. For verifying the suggested viewpoint above, we performed a measurement of the relationship between the number of cycles and the shape recovery ratio Figure 10d. Interestingly, we found that the shape recovery ratio of SMWs decreased sharply before three deformation cycles and stabilizes after the fourth deformation cycle. The result is mainly owing to the plastic deformation of the wood reaching the limit after several deformation cycles. In order to further explore the influence of wood plastic deformation on shape recovery ratio, we researched the relationship between first figuration angle degree and the shape recovery ratio of SMW as shown in Figure 10c, which indicated the shape recovery ratio of SMW decreases as the first figuration angle degree increases, however, the shape recovery ratio of c-PCL basically maintains a constant value about 100% at the same time. On the other hand, it can be found that shape recovery ratio sharply decreases when first figuration angle is greater than 60 degrees, and SMW broken at close to 100 degrees, and no cracks were found on the curving surface before that. This significant downtrend can be attributed to the wood yield phenomenon, when the inner stress exceeds the elastic limit as illustrated in Figure 11. According to the above analysis, we think reducing the porosity of SMW may increase the shape recovery ratio, which needs to optimize the impregnation process.

The shape recovery properties (both the shape recovery rate and the final recovery ratio) also depended on the environment temperature within a limited time (within 30 min). As shown in Figure 10b, the final shape recovery ratio shows a rising tendency with the increase of environment temperature for a heating time (30 min). When the temperature is higher than about 70 °C, the final shape recovery ratio of SMW reaches a relatively stable ratio that is varying, responding to different samples on account of the biological characteristics of natural wood. In addition, compared with 50 °C and 40 °C, when the environment temperature is higher than 60 °C, the shape recovery rate is almost the same in the initial stage and the shape recovery ratio can reach maximum within about 5 min.

Section summary: According to above-mentioned conclusions, it is clearly that the wood-PCL composite material had possessed shape memory effect, and showed outstanding shape fixity ratio of nearly 99%.

## 4. Conclusions

In this paper, we fabricated heat-triggered shape memory wood composite by three-step method using balsa wood and PCL, in which PCL would form chemical crosslinking structure inside wood after curing process. For softening wood and increasing porosity, it is prerequisite to remove lignin and hemicellulose properly, and which also can improve the dispersity of PCL in the three-dimension mesoporous structure of wood.

The formation of hydrogen bond between cellulose fibers and PCL molecular chain interfaces endows SMW with excellent physical stability and outstanding mechanical strength. Especially the tensile strength of SMW (10.68 MPa) at room temperature is 805% higher than natural wood along the direction of cross section. The fabricated shape memory wood exhibits excellent shape fixity ratio of about 96%, but the shape recovery ratio is relatively low and decreases as the number of deformation cycles increases. Both SMW and c-PCL have similar thermal properties, such as analogous DSC and DMA variation tendency, which indicates good compatibility and coordination between wood and PCL.

Further, we would aim to investigate the effect of weight concentration and cross-linking density of SMP on the properties of SMW. In addition, PCL and wood have similar biological properties such as biocompatibility and biodegradability, and is also an environmentally friendly and pollution-free organic material. These merits can further enlarge the adaptive application territory of SMW such as biomimetic architecture, self-healing wood veneering, and intelligent furniture.

## Figures and Tables

**Table 1:** The weight concentration and the volume filling ratio of SMP in SMW; the swelling (contract) ratio of SMW in the longitudinal (width), radius (length), and tangential (thickness) direction defined as SR-L, CR-R, and SR-T, respectively, compared with LRW.

No.	The Weight of LRW (g)	The Weight of SMW (g)	*W_cSMP_* (%)	*V_rSMP_* (%)	SR-L (%)	CR-R (%)	SR-T (%)
**S1**	0.213	1.428	570.42	87.41%	0.11%	1.83%	4.46%
**S2**	0.235	1.491	534.47	87.70%	1.92%	0.11%	6.44%
**S3**	0.198	1.355	584.34	85.17%	2.37%	−0.81%	2.97%
**S4**	0.227	1.406	519.38	84.18%	1.35%	0.41%	3.96%
**S5**	0.215	1.432	566.05	88.22%	1.58%	1.57%	3.96%
**P_LRW_ = 93.27% ρ_PP_ = 1.129 g/mL**							

**Table 2:** The Gel Contents of SMW, crosslinking-PCL (c-PCL), and The Extractive Content of Balsa Wood.

Name	Gel Content	*m*_0_ (g)	*m* (g)
**SMW**	56.83%	1.426	0.892
**c-PCL**	61.53%	2.620	1.612
**LRW**	−	0.535	0.510

*m*_0_: The initial weight before extraction; *m*: The residual weight after extraction; the weight of lignin-removed wood (LRW) scaffold in SMW = 0.212 g.
